# Current perspectives and trend of ferroptosis in head and neck cancer: a bibliometric analysis

**DOI:** 10.3389/froh.2025.1601962

**Published:** 2025-08-08

**Authors:** Jin Pan, Yi Zhang, Binbin Zhao, Wei Shao, Xin Chen

**Affiliations:** ^1^ Key Laboratory of Oral Diseases Research of Anhui Province, College & Hospital of Stomatology, Anhui Medical University, Hefei, China; ^2^ Anhui Provincial Laboratory of Pathogen Biology, Department of Microbiology and Parasitology, School of Basic Medical Sciences, Anhui Medical University, Hefei, Anhui, China

**Keywords:** ferroptosis, head and neck cancers, cancer therapy, cell apoptosis, bibliometric analysis

## Abstract

**Background:**

Ferroptosis, a form of regulated cell death driven by iron dependency, is also referred to as iron-mediated cell death. In recent years, ferroptosis has garnered considerable research interest as a distinct cell death mechanism, primarily characterized by excessive intracellular iron accumulation and the formation of lipid peroxides. Ferroptosis is intricately linked to the initiation and progression of a wide spectrum of diseases, notably cancers, neurodegenerative disorders—including Alzheimer's and Parkinson's diseases—and cardiovascular conditions. Despite growing interest in ferroptosis within cancer research, systematic analyses and comprehensive visualizations of research hotspots, leading contributors, and emerging trends—particularly in the context of head and neck cancers—remain limited.

**Materials and methods:**

This study conducted a bibliometric analysis using data retrieved from the Web of Science Core Collection, covering the period from January 1, 2016, to March 20, 2025. Bibliometric mapping and visualization were performed using VOSviewer and CiteSpace.

**Results:**

A total of 110 publications were identified across 19 countries. China contributed the highest number of publications (84 articles; 76.36%), followed by South Korea (14 articles; 12.73%) and the United States (6 articles; 5.46%). *Nature*, *Cell*, and *Cancer Letters* emerged as the leading academic journals publishing on ferroptosis in the context of head and neck cancers. Emerging high-frequency keywords—such as “expression” “cell death” “ferroptosis” “inhibition” “metabolism” and “therapy”—highlight evolving research frontiers in this field.

**Conclusion:**

This study presents a comprehensive overview of recent trends and advances in ferroptosis research within the context of head and neck cancer, delineating key research frontiers and emerging thematic areas. The findings offer valuable insights and serve as a useful reference for researchers pursuing work in this domain.

## 1 Introduction

Head and neck cancer (HNC) encompasses a group of malignancies arising in anatomical regions such as the oral cavity, pharynx, larynx, nasal cavity, paranasal sinuses, ears, and salivary glands (1). The prognosis of HNC is determined by multiple factors, including histological type, tumor stage, patient age, and general health status. HNC is clinically heterogeneous, exhibiting considerable variation in etiology, clinical manifestations, therapeutic strategies, and outcomes. HNC ranks among the most prevalent malignant tumors worldwide (2). According to the World Health Organization (WHO), HNC constitutes the sixth most commonly diagnosed cancer globally. Incidence rates vary by region and country; however, they are consistently higher in males than in females, with tobacco use and alcohol consumption identified as the primary risk factors (3). Management of HNC typically involves a multidisciplinary approach, with therapeutic decisions guided by tumor type, clinical stage, and patient health status. Standard treatment modalities include surgery, radiotherapy, chemotherapy, and immunotherapy (4). Early diagnosis and timely intervention are critical for improving survival outcomes. However, the overall survival rate remains suboptimal, particularly for patients diagnosed at advanced stages (5).

Ferroptosis is a recently identified form of regulated cell death characterized by iron dependency and lipid peroxidation (6). In contrast to classical cell death modalities—including apoptosis, necrosis, and autophagy (7),—ferroptosis is predominantly driven by iron-mediated oxidative stress that induces lipid peroxidation of cellular membranes, culminating in cell death (8). The mechanistic basis of ferroptosis can be delineated across several interconnected pathways: (1) Iron accumulation: The excessive accumulation of iron inside cells is a key factor in ferroptosis. Cells absorb iron via transferrin (TF) and store it in ferritin. Iron overload may be caused by overactivation of transferrin receptors, reduced expression of ferritin, or other factors (9); (2) Lipid peroxidation: Lipid peroxidation is the hallmark process of ferroptosis. Particularly, polyunsaturated fatty acids (PUFAs) in the cell membrane are highly prone to peroxidation, leading to the generation of peroxides that damage the cell membrane structure and result in cell death (10); (3) GPX4 inhibition: Glutathione peroxidase 4 (GPX4) is one of the main antioxidant enzymes, capable of reducing lipid peroxides and protecting cells from lipid peroxidation-induced damage. When GPX4 activity is impaired, lipid peroxidation is exacerbated, leading to ferroptosis (11); (4) Reactive oxygen species (ROS) generation: Iron ions catalyze the production of excessive ROS through the Fenton and Haber-Weiss reactions. These ROS further amplify lipid peroxidation and promote cell death (12).

Head and neck tumors are marked by pronounced heterogeneity and inherent resistance to conventional therapies. Conventional treatments—including radiotherapy and chemotherapy—often suffer from suboptimal efficacy and high rates of recurrence. Ferroptosis is not only implicated in the initiation and progression of head and neck cancers, but also shows potential as a novel therapeutic target for overcoming treatment resistance and improving clinical outcomes. Therefore, elucidating the regulatory mechanisms of ferroptosis in head and neck tumors—and its crosstalk with the immune microenvironment and metabolic reprogramming—is critical for the development of innovative anticancer strategies and the realization of personalized precision medicine. In recent years, ferroptosis has emerged as a promising research frontier in the therapeutic landscape of head and neck cancer (13). In this article, we evaluated recent advancements in the study of ferroptosis in head and neck tumors and employed bibliometric techniques to examine emerging research trends. Using data from the Web of Science Core Collection (2016–2025), we conducted a comprehensive visual analysis encompassing publication volume by year, geographical and institutional contributions, authorship patterns, source journals, keyword co-occurrence, and co-citation networks. This study provides a structured overview of the intellectual landscape of this field, offering valuable guidance for both established researchers and newcomers. By identifying influential contributors, topical hotspots, and evolving research directions, the visualized results serve as a foundation for more targeted research planning and foster the development of future investigations in a systematic and accessible manner.

## 2 Materials and methods

### 2.1 Data collection

**Figure 1:**
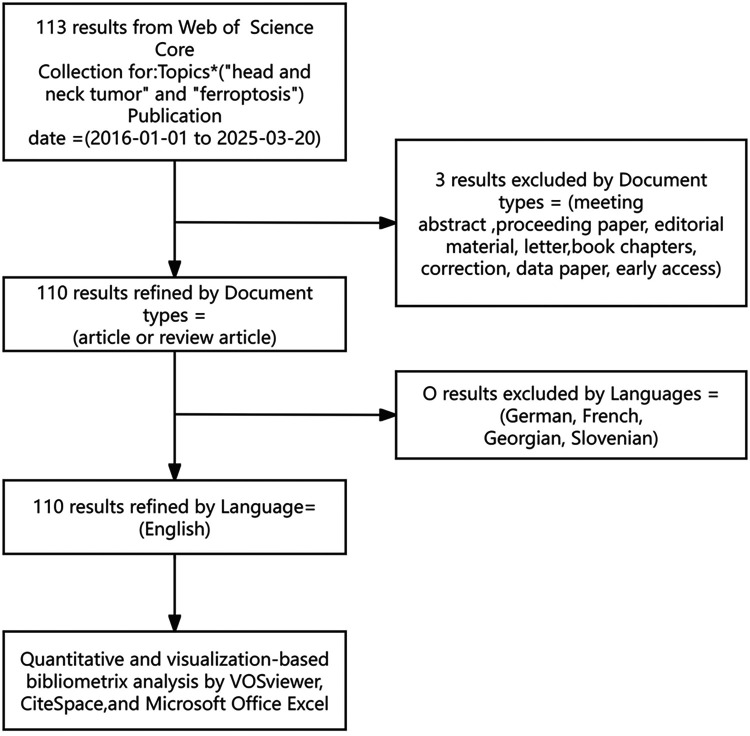
Publications screening flowchart.

The Web of Science (WOS) Core Collection is a globally recognized multidisciplinary academic database that provides access to high-quality scholarly literature from a wide range of disciplines. It is one of the most extensively used resources in academia, supporting literature retrieval, citation analysis, research evaluation, and the exploration of scholarly trends. Additionally, WOS serves as a primary source for bibliometric analyses by enabling the export of general statistical data compatible with various visualization tools. Owing to its breadth and reliability, WOS remains the most commonly employed database in bibliometric research. In this study, literature was retrieved from WOS using the search string: TS = (head and neck tumor) AND (ferroptosis). The search spanned from January 1, 2016, to March 20, 2025. Only original articles and review articles published in English were included. Exclusion criteria encompassed conference abstracts, editorials, proceedings papers, letters, early access publications, corrections, retracted publications, notes, retractions, expressions of concern, and other non-article document types. Retrieved records were saved in “plain text” format and exported with “full citation records”; the resulting files were named *download.txt*. A detailed flowchart outlining the literature screening and selection strategy is provided in Figure 1.

### 2.2 Visualized analysis

VOSviewer (version 1.6.20.0) and CiteSpace (version 6.3.1) were used to import and analyze bibliometric data retrieved from the Web of Science Core Collection. These tools are widely adopted for generating visual representations of scientific landscapes. In this study, key bibliometric indicators—including co-authorship, co-occurrence, and co-citation—were employed to map the structure and dynamics of research activity. Co-authorship analysis was conducted to explore collaborative networks among authors, institutions, and countries. Co-occurrence analysis, based on keyword frequency and proximity, was applied to identify thematic linkages and research hotspots. Citation and co-citation analyses were performed to assess the intellectual influence and interconnectedness of cited literature. The primary variables collected for analysis included authors, institutional affiliations, countries or regions, source journals, keywords, and references.

VOSviewer is a specialized software tool designed for constructing and visualizing bibliometric networks, and it is widely used in academic research for data visualization purposes (14). The software supports the processing of bibliographic data from multiple databases—particularly those in the scientific domain—to explore relationships among authors, keywords, journals, institutions, and citations. Its core functionalities include network visualization, clustering analysis, textual data mining, interactive graphical outputs, and flexible options for data import and export (15).

CiteSpace is a powerful tool for bibliometric analysis and the visualization of scientific knowledge structures. It is widely employed in scholarly research to uncover emerging trends, trace the evolution of knowledge, and identify critical topics within academic domains. CiteSpace generates visual representations of academic networks by analyzing citation patterns, co-citation clusters, keyword co-occurrence, and other bibliometric indicators, thereby highlighting major developments and research frontiers in a given field. In addition, the platform offers an intuitive graphical interface that facilitates the interpretation of complex bibliometric data by allowing users to visually explore structural and temporal patterns (16).

## 3 Results

### 3.1 The global trends in publication output and citations

**Figure 2:**
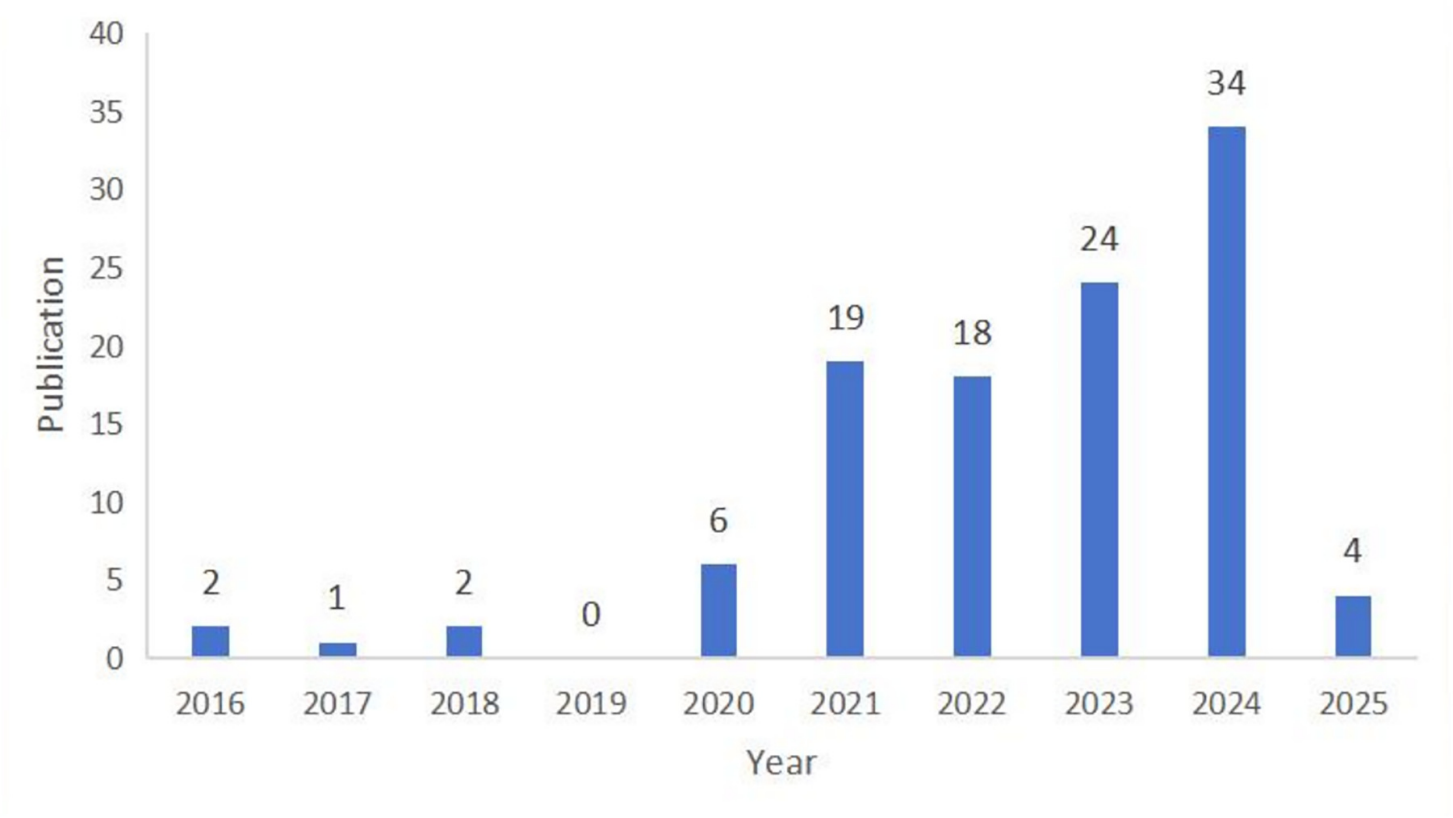
Global publication output and citation trend on head and neck tumors and ferroptosis from 2016 to 2025.

A total of 110 articles related to ferroptosis and head and neck tumors were identified in the Web of Science database. Figure 2 presents the global trends in publication output and total citations from 2016 to 2025. Notably, research on ferroptosis in the context of head and neck tumor treatment has expanded rapidly, particularly over the past four years, during which approximately 90% of the publications were produced. The annual number of articles increased from just 2 in 2016 to 34 in 2024, with a marked acceleration in output observed after 2021. As of the retrieval date, the collected literature had been cited a total of 3,794 times, with an average of 35.79 citations per article. Current research continues to concentrate on exploring the therapeutic potential of ferroptosis in the treatment of head and neck tumors.

### 3.2 Distribution of countries

Publications on ferroptosis in the context of head and neck tumors originate from 19 different countries or regions. As illustrated by the global productivity map, the majority of these studies have been published in countries across Asia and North America. The top ten most productive countries are listed in Table 1. China leads by a substantial margin, contributing 84 publications (76.36%), followed by South Korea with 14 articles (12.73%) and the United States with 6 articles (5.46%).

**Table 1:** Top 10 productive countries/regions related to head and neck tumors and ferroptosis.

Rank	Country	Number
1	China	82
2	South Korea	14
3	USA	6
4	France	2
5	Germany	1
6	Russia	1
7	New Zealand	1
8	Belgium	1
9	Brazil	1
10	Italy	1

### 3.3 Distribution of institutions

**Figure 3:**
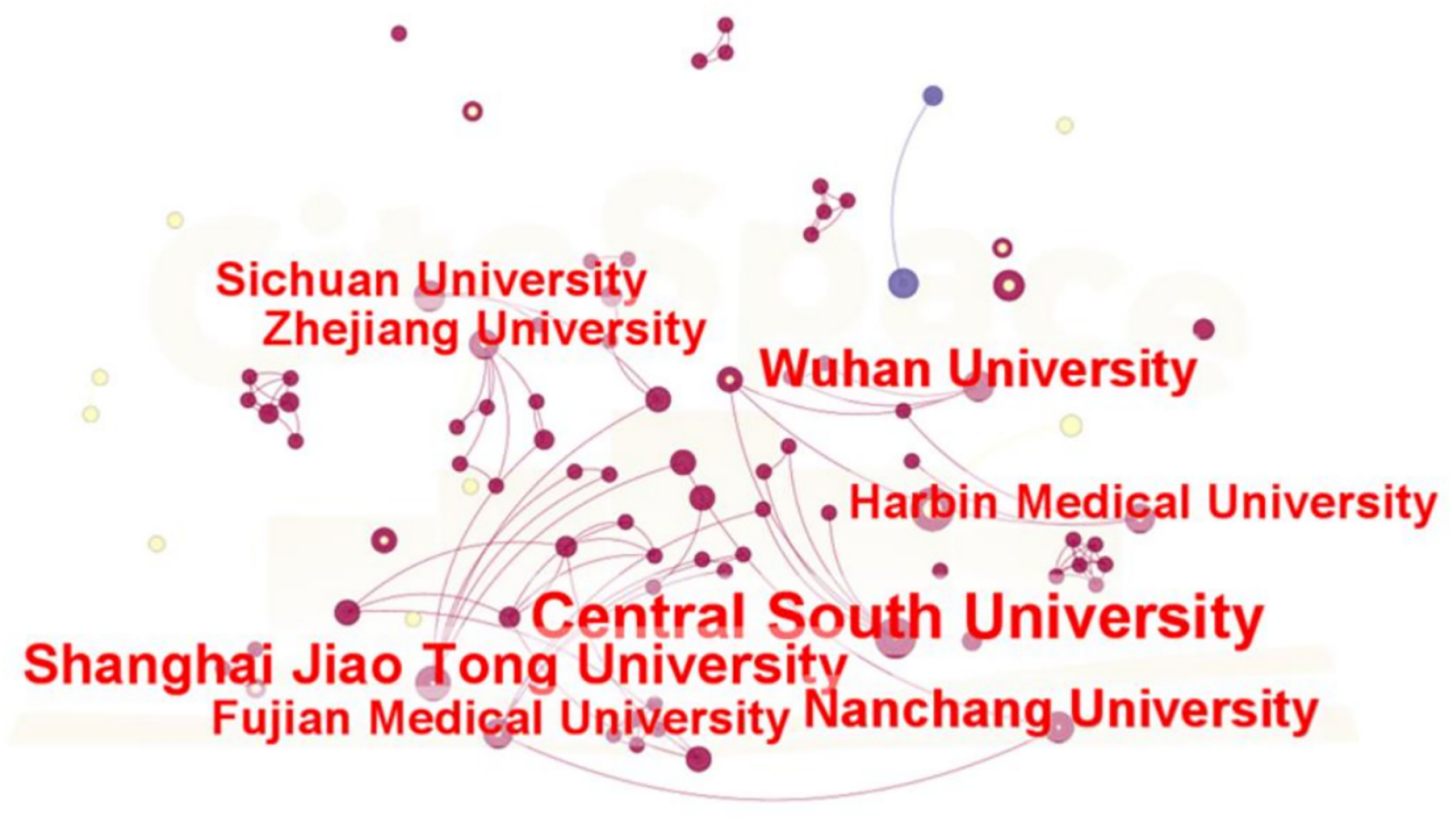
The institutions' collaboration network visualization map generated by Citespace software.

A total of 136 institutions have contributed to publications on ferroptosis in head and neck tumors. Table 2 presents the top ten institutions ranked by publication volume. China hosts a significant number of research-active institutions in this domain, with the majority of contributions coming from Baochuan Tea University, Central South University, and Shanghai Jiao Tong University. Collectively, the top ten institutions account for 54 publications, representing 50.94% of the total output. To examine patterns of institutional collaboration, a network visualization map was generated using CiteSpace. As shown in Figure 3, the collaboration network comprises 136 nodes and 184 links, indicating a relatively dense structure. Institutional collaboration appears to be more extensive than inter-country collaboration. Notably, Central South University maintains active research partnerships with multiple academic institutions across China.

**Table 2:** The top 10 productive institutions ranked by the numbers of publications.

Rank	Institution	Number
1	Cha university	9
2	Cent south university	8
3	Shanghai jiao tong university	6
4	Wuhan university	5
5	Nanchang university	5
6	Sun yat sen university	5
7	Zhejiang university	4
8	Sichuan university	4
9	China med university	4
10	University ulsan	4

### 3.4 Authors and co-cited authors

**Figure 4:**
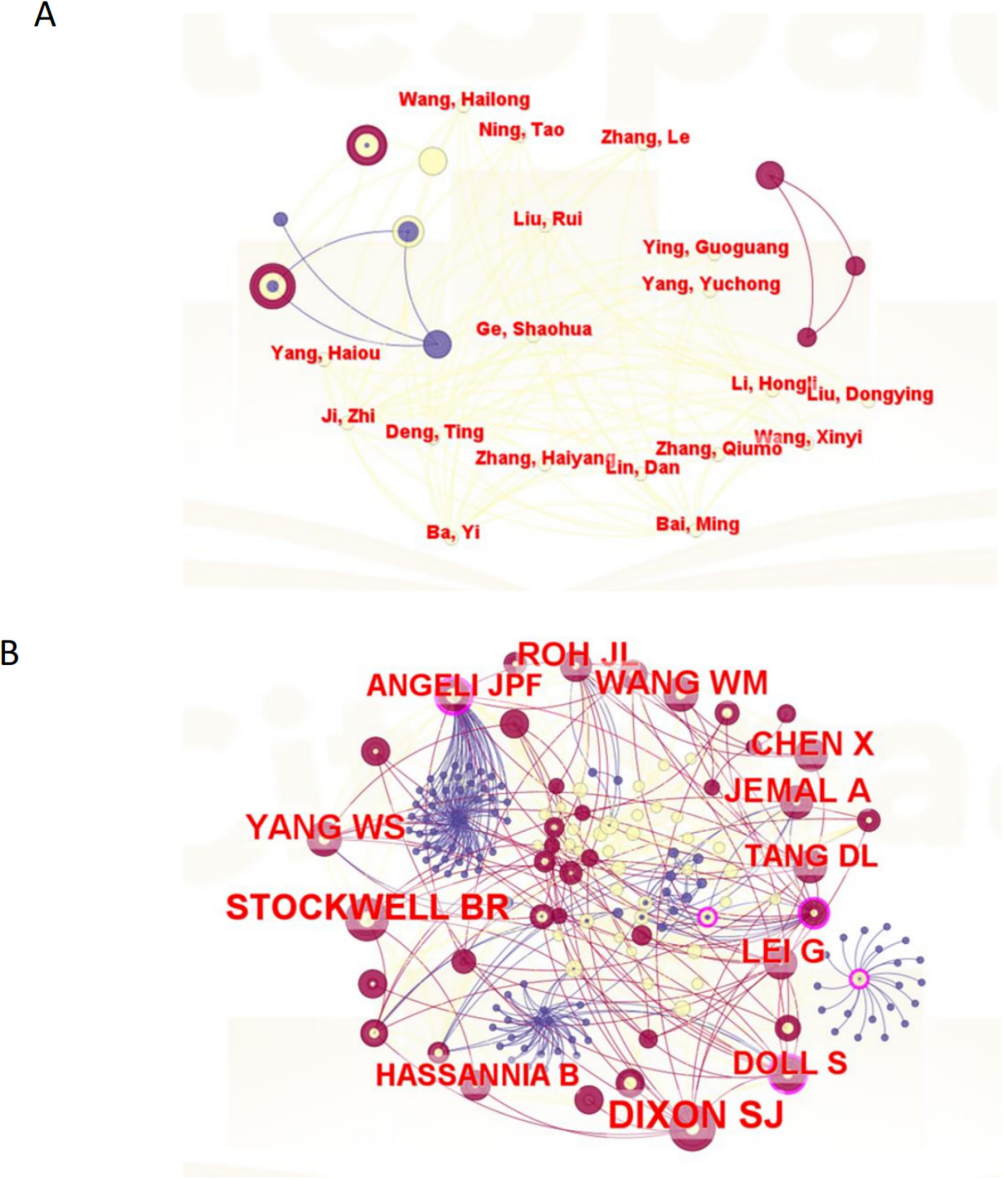
The visualization map of co-authorship **(A)** and co-citation **(B)** analyses of authors carried on CiteSpace.

A total of 660 authors have contributed to publications on ferroptosis in head and neck tumors, and 2,526 authors have been co-cited within this body of literature. Tables 3, 4 present the top ten most productive authors by publication count and the top ten most frequently co-cited authors, respectively. The author collaboration network is visualized in Figure 4. Each node represents an author, while links between nodes indicate co-authorship; thicker lines denote stronger collaborative ties, and node colors reflect the year of collaboration. The structure of the network is quantitatively assessed using two key metrics: the modularity Q value, which measures the degree of cluster separation, and the silhouette value, which reflects the internal cohesion of clusters. In this study, the modularity Q value is 0.906, and the silhouette score is 0.7, indicating that the author collaboration network exhibits well-defined clusters with a high degree of internal consistency and minimal overlap between groups. Authors tend to collaborate more closely within their own clusters than across clusters. Node importance is further evaluated through betweenness centrality (BC), which captures the extent to which a node acts as a bridge connecting different parts of the network. A BC value of 0.1 or above typically signals a key intermediary. Notably, Brent R. Stockwell (BC = 0.75), Scott J. Dixon (BC = 0.72), and Sebastian Doll (BC = 0.68) exhibit high centrality scores, identifying them as pivotal “bridge figures” linking distinct research themes. These scholars have played influential roles in shaping the interdisciplinary development of ferroptosis research in head and neck tumors. In addition, author impact was assessed using the h-index, which quantifies both the productivity and citation impact of an individual researcher. As shown in Table 3, Roh Jong-Lyel leads with 13 publications, followed by Lee Jaewang (10 publications) and Shin Daiha (6 publications), indicating their prominent contributions to the field.

**Table 3:** The top 10 most productive authors related to head and neck tumors and ferroptosis.

Rank	Author	Number	Citations
1	Jong-Lyel Roh	13	1,753
2	Jaewang Lee	10	1,017
3	Daiha Shin	6	1,502
4	Ji Hyeon You	5	423
5	Kim eunhye	4	1,318
6	Li jing	4	18
7	Ming-Shou Hsieh	3	8
8	Syahru Agung Setiawan	3	8
9	Chi-Tai Yeh	3	8
10	Wang qi	3	8

**Table 4:** The top 10 most productive co-cited authors related to head and neck tumors and ferroptosis.

Rank	Cited author	Number
1	Scott J Dixon	54
2	Brent R Stockwell	38
3	Wan Seok Yang	35
4	Andre Gemal	29
5	Weimin Wang	29
6	Jong-Lyel Roh	29
7	Guang Lei	28
8	Xin Chen	27
9	Sebastian Doll	24
10	Daolin Tang	23

### 3.5 Distribution of source journals

**Figure 5:**
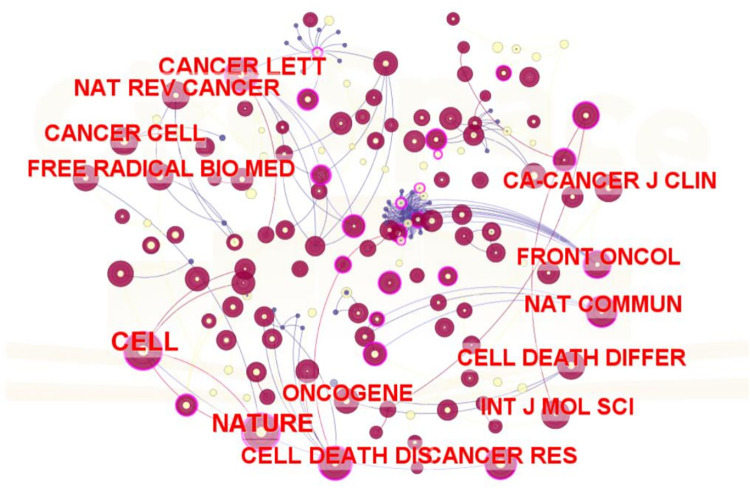
The visualization map of co-cited journals analyses of authors carried on CiteSpace.

Research on ferroptosis and head and neck tumor treatment has been disseminated across 267 academic journals. The relationship between them is shown in Figure 5. Among them, *Cell* (*n* = 82), *Nature* (*n* = 75), and *Cancer Letters* (*n* = 61) rank as the top three sources by publication volume. Table 5 summarizes the top ten co-cited journals in this field. Notably, 40% (4 out of 10) of these journals are based in the United Kingdom, while 20% (2 out of 10) are published in the United States, highlighting the geographical distribution of high-impact publishing venues in this research domain.

**Table 5:** The top 10 co-cited journals related to head and neck tumors and ferroptosis.

Rank	Cited journal	Number
1	Cell	82
2	Nature	75
3	Cancer letter	61
4	Cancer research	59
5	Nature communications	57
6	Cell death & disease	56
7	Nature reviews cancer	52
8	Cancer cell	50
9	Cell death and differentiation	50
10	International Journal of Molecular Sciences	49

### 3.6 Analysis of top co-cited references

**Figure 6:**
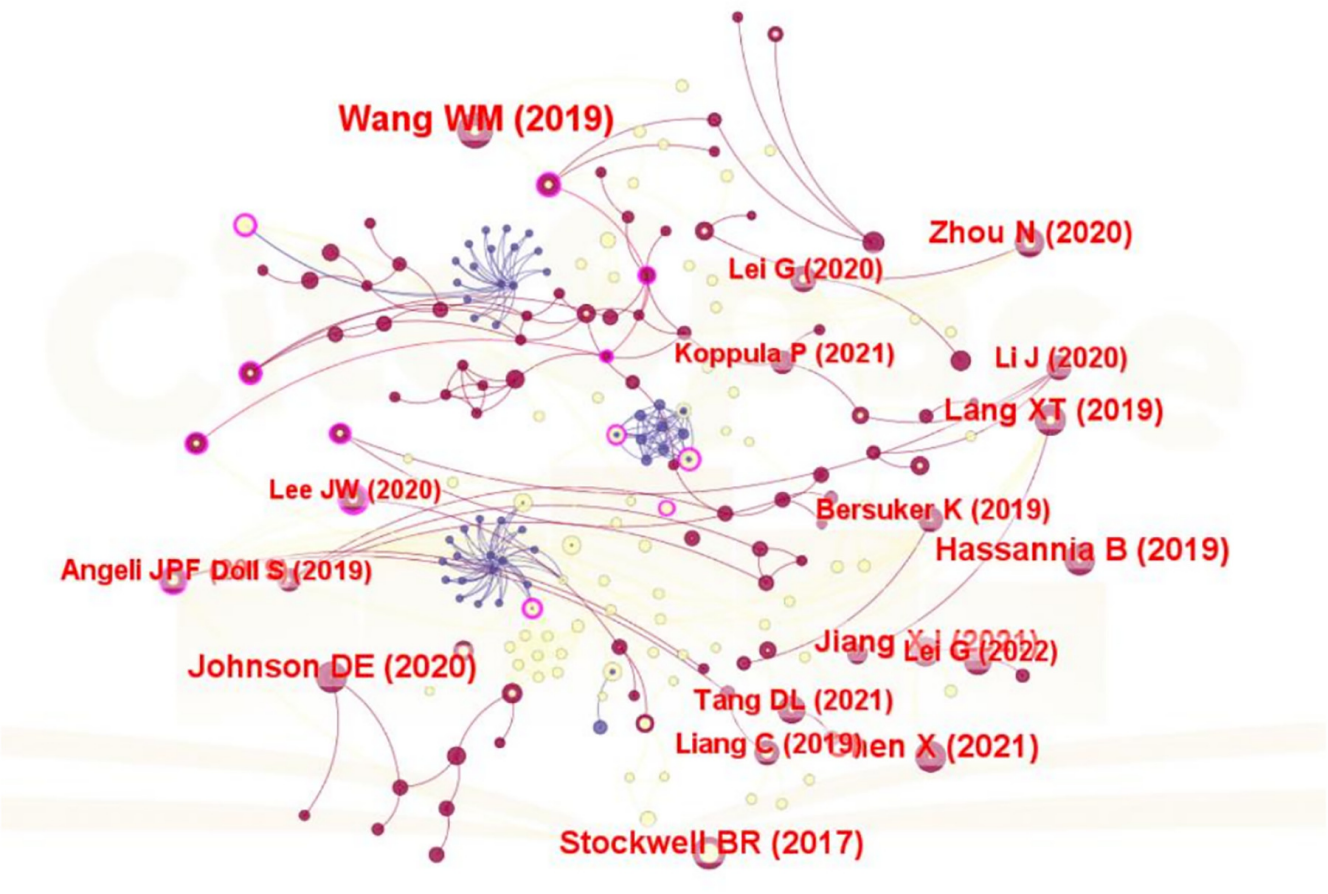
The visualization map of co-cited references analyses of authors carried on CiteSpace.

Over the past decade, a total of 40,719 references have been cited within the literature on ferroptosis and head and neck tumors. As shown in Table 6, all of the top ten most frequently co-cited papers have been cited more than 600 times, with the most highly co-cited paper reaching 3,255 citations. A total of 292 publications were included in the construction of the co-citation network, as visualized in Figure 6. According to the network structure, *Angeli JPF, 2019* exhibits strong co-citation linkages with *Stockwell BR, 2017*, *Mou YH, 2019*, and *Gao MH, 2019*, indicating a shared thematic foundation and sustained academic relevance.

**Table 6:** Top 10 co-cited references on research of head and neck tumors and ferroptosis.

Rank	Title	Journal	First author	Year	Citations
1	CD8 + T cells regulate tumour ferroptosis during cancer immunotherapy	Nature	Wang weimin	2019	1,787
2	Head and neck squamous cell carcinoma	NATURE REVIEWS DISEASE PRIMERS	Johnson, Daniel E.	2020	1,701
3	Broadening horizons: the role of ferroptosis in cancer	NATURE REVIEWS CLINICAL ONCOLOGY	Chen, Xin	2021	1,622
4	Targeting Ferroptosis to Iron Out Cancer	CANCER CELL	Hassannia, Behrouz	2019	1,605
5	Ferroptosis: mechanisms, biology and role in disease	NATURE REVIEWS MOLECULAR CELL BIOLOGY	Jiang, Xuejun	2021	3,255
6	Targeting ferroptosis as a vulnerability in cancer	NATURE REVIEWS CANCER	Lei, Guang	2022	1,125
7	Ferroptosis: past, present and future	CELL DEATH & DISEASE	Li, Jie	2020	2,260
8	Radiotherapy and Immunotherapy Promote Tumoral Lipid Oxidation and Ferroptosis via Synergistic Repression of SLC7A11	CANCER DISCOVERY	Lang, Xueting	2019	682
9	Ferroptosis: molecular mechanisms and health implications	CELL RESEARCH	Tang, Daolin	2021	1,996
10	The CoQ oxidoreductase FSP1 acts parallel to GPX4 to inhibit ferroptosis	Nature	Bersuker, Kirill	2019	2,102

### 3.7 Keywords analysis of research hotspots

**Figure 7:**
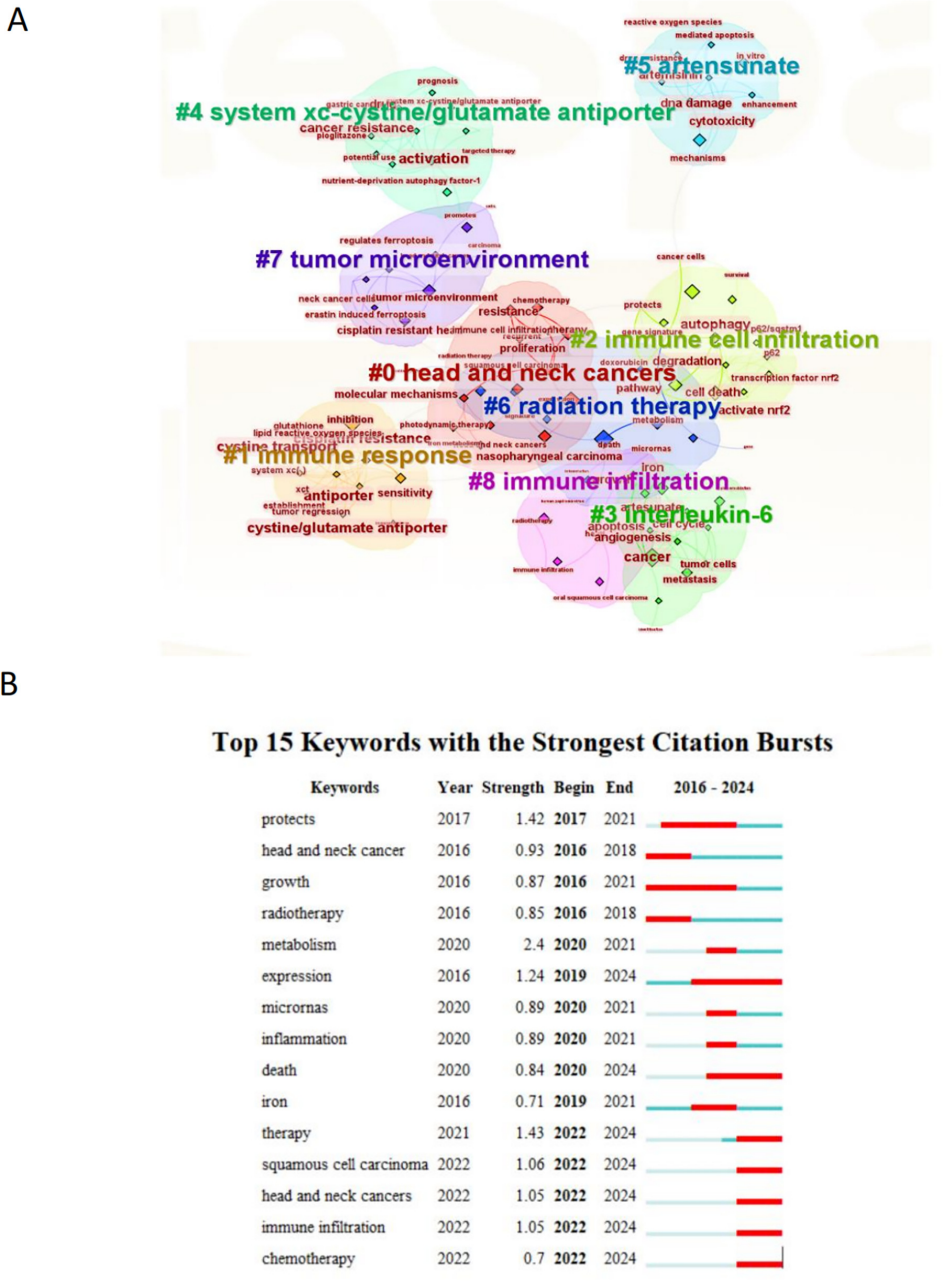
CiteSpace visualization map of cluster view **(A)** and top 15 keywords with the strongest citation bursts **(B)**.

Using CiteSpace, the top 90 keywords from the current body of research were extracted and subjected to clustering analysis, as shown in Figure 7. This figure highlights both well-established themes and underexplored areas within the domain of ferroptosis and head and neck tumor research. The clustered keywords are grouped into nine distinct thematic clusters, each represented by a different color. Frequently occurring terms include “expression,” “death,” “cancer,” “inhibition,” and “cell death,” reflecting the prevailing research focus. Another important metric for identifying research frontiers and emerging trends is keyword burst intensity, which denotes the sudden increase in keyword usage over time. As shown in Figure 7, several terms exhibit strong and sustained citation bursts, including “expression” (2019–2024), “death” (2020–2024), “therapy” (2022–2024), “chemotherapy” (2022–2024), “immune infiltration” (2022–2024), and “head and neck cancer” (2022–2024). These extended burst periods, which continue through 2024, indicate that these topics have recently garnered significant scholarly attention and may represent ongoing or emerging research frontiers.

We further filtered 93 high-frequency keywords and conducted a clustering analysis using CiteSpace (Figure 7). Several distinct clusters were identified, each representing a specific research direction within the field of ferroptosis and head and neck tumors. The top three clusters, visualized in Figure 7, are characterized by the following themes: the red cluster contains keywords such as *head and neck cancers*, *resistance*, and *proliferation*; the yellow cluster includes terms like *immune response*, *cisplatin resistance*, and *antiporter*; and the green cluster comprises keywords such as *immune cell infiltration*, *cell death*, and *degradation*. These clusters reflect the current thematic structure of research and underscore the multidimensional nature of ongoing investigations.

**Figure 8:**
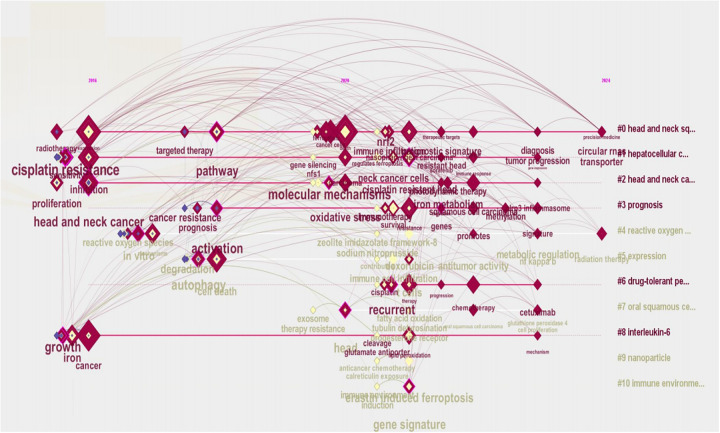
Timeline view of eywords carried on CiteSpace.

A keyword timeline map was generated to examine the temporal evolution of research hotspots, as illustrated in Figure 8. The primary cluster centers around *head and neck squamous cell carcinoma*, suggesting that this area has received the most sustained and concentrated research attention over time. In the early phase (2016–2019), investigations primarily focused on the fundamental mechanisms of ferroptosis and cellular responses, marking the initial exploratory stage of the field. During the middle period (2020–2022), research efforts shifted toward the integration of ferroptosis with applied topics, including tumor drug resistance and regulation of the immune microenvironment. In the most recent period (2023–2024), the emergence of terms such as *diagnostic signature*, *tumor progression*, *precision medicine*, and *circular RNA* indicates a growing convergence of ferroptosis research with advanced fields such as precision oncology and epigenetics.

## 4 Discussion

This study retrieved 110 publications on ferroptosis and head and neck cancers from the Web of Science Core Collection, covering the period from 2016 to 2025. A notable increase in annual publication output has occurred since 2021, with a marked surge over the past two years. Over the past nine years, China has emerged as the leading contributor to this research field, accounting for the majority of publications; notably, eight of the top ten most productive institutions are based in China. North American countries, particularly the United States, have also made substantial contributions. Extensive collaborative research is evident across both countries and institutions. Despite the high incidence of head and neck cancers in Central and South Asia—largely attributable to widespread betel nut consumption—research focusing on the role of ferroptosis in these regions remains limited. This geographic imbalance highlights a potential gap in the literature that warrants further investigation in future studies (17).

Among the top ten ranked journals, *Nature*, *Cell*, and *Cancer Letters* lead in terms of the number of publications on ferroptosis in head and neck cancers and are also among the most frequently co-cited sources. The majority of research in this field is published in journals specializing in molecular biology, immunology, and medical or clinical sciences. This distribution suggests that current studies have achieved notable progress in both fundamental mechanistic research and translational or clinical applications.

From an authorship perspective, Roh Jong-Lyel of Asan Medical Center is the most prolific contributor to the field, with a total of 13 publications. He is recognized as one of the pioneering figures in the study of ferroptosis in head and neck tumors. In terms of co-citation impact, Dixon S.J. ranks first, having conducted foundational work on the cell biology of ferroptosis (18). Stockwell B.R. and Yang W.S. are the second- and third-most frequently co-cited authors, respectively. Stockwell's research has focused on the induction of ferroptosis by polyunsaturated phosphatidylcholine species (PC-PUFA2s) across various cancer cell lines (19), while Yang has extensively explored the molecular regulatory landscape of ferroptosis (20).

Co-cited literature refers to references that are cited simultaneously by multiple publications. Such references often represent foundational studies or highlight key research hotspots within a given field. In this bibliometric study, the top ten most frequently co-cited references were identified. Based on a literature review, these references can be broadly categorized into two groups: three clinical trial articles focused on the therapeutic potential of ferroptosis (21–23), and seven review articles that provide comprehensive overviews of ferroptosis mechanisms and applications (3, 6, 24–28). Collectively, these works offer in-depth insights into the molecular pathways by which ferroptosis suppresses tumor growth, as well as perspectives on its potential clinical translation in cancer therapy.

Keyword analysis provides a rapid means of capturing the distribution and evolution of research hotspots in the field of ferroptosis and head and neck cancers. Frequently occurring terms such as *expression*, *immune response*, *cancer*, and *inhibition* reflect the primary thematic directions of current research. Keyword clustering analysis further identified three major research categories: the *head and neck cancer* cluster, the *immune response* cluster, and the *immune cell infiltration* cluster. From a research trend perspective, ferroptosis has emerged as a promising therapeutic target, particularly in cancer treatment. Since 2016, a growing number of studies have explored strategies to induce ferroptosis for the treatment of head and neck cancers. These include the use of ferroptosis inducers or iron metabolism regulators to inhibit tumor growth (29), enhance sensitivity to radiotherapy and chemotherapy (30), and overcome drug resistance (31). Such approaches offer novel insights for clinical oncology. More recently, ferroptosis research has extended into cutting-edge areas such as precision-targeted therapies (32), molecular biomarker screening (33), and combination therapy models (34). Collectively, these advances signal a shift in ferroptosis research from foundational mechanistic studies toward translational and clinical applications. Increasing recognition of ferroptosis as a modulator of immune responses further underscores its potential as a multifaceted strategy in cancer treatment, particularly in head and neck oncology.

Ferroptosis is regulated through multiple interconnected pathways, including iron metabolism, lipid peroxidation, and glutathione depletion. Future research should leverage high-throughput technologies—such as single-cell sequencing—to gain deeper mechanistic insights into ferroptosis regulation in head and neck squamous cell carcinoma (HNSCC), thereby establishing a theoretical basis for the development of novel therapeutic strategies. Although ferroptosis inducers such as Erastin and RSL3 have demonstrated antitumor efficacy in various cancer types, their application in HNSCC remains largely unexplored. Further efforts should be directed toward identifying and optimizing ferroptosis inducers with enhanced specificity and safety profiles tailored to HNSCC. Moreover, ferroptosis may exhibit synergistic effects when combined with conventional therapies, including radiotherapy, chemotherapy, and immunotherapy. Investigating such combination strategies may improve overall treatment efficacy and help overcome therapeutic resistance. From a clinical perspective, a subset of HNSCC patients—particularly those resistant to standard treatments—may be more responsive to ferroptosis-based therapies. Clinicians are encouraged to monitor biomarkers such as GPX4 and SLC7A11 to identify candidates for ferroptosis-targeted interventions, thereby facilitating more precise and individualized treatment planning.

This study has several limitations. First, all data were sourced exclusively from the Web of Science Core Collection, which may have led to the omission of relevant publications indexed in other databases. Second, only English-language articles were included, introducing the possibility of language and selection bias. Third, literature published in the final months beyond the cut-off point in 2025 was not captured, which may slightly affect the accuracy of trend projections.

To the best of our knowledge, this is the first comprehensive bibliometric analysis of research on ferroptosis and head and neck cancers conducted over the past decade. The rapid increase in publication output reflects growing global interest in this emerging field. China has played a leading role by contributing a large number of high-impact publications and fostering high-quality research. Moreover, extensive collaborations between countries and institutions have further advanced the field. In summary, the application of ferroptosis in head and neck cancer treatment demonstrates significant potential, with encouraging progress observed in both basic research and clinical translation.

## Data Availability

The original contributions presented in the study are included in the article/Supplementary Material, further inquiries can be directed to the corresponding authors.
